# Jejuno-jejunal intussusception caused by a percutaneous endoscopic gastrojejunostomy tube in a pediatric patient: A case report

**DOI:** 10.1097/MD.0000000000019888

**Published:** 2020-04-17

**Authors:** Toshihiko Kakiuchi, Aiko Nakayama, Junichi Nojiri, Takeshi Yamanouchi, Muneaki Matsuo

**Affiliations:** a Department of Pediatrics; b Department of Radiology, Faculty of Medicine, Saga University; c Department of Pediatric Surgery, Saga-Ken Medical Center Koseikan, Saga, Japan.

**Keywords:** duodenum, intestinal malrotation, percutaneous endoscopic gastrojejunostomy

## Abstract

**Rationale:**

Although percutaneous endoscopic gastrojejunostomy (PEG-J) tubes are believed to reduce the side effect of aspiration, cautious catheter management is required. Intussusception is a serious complication of these tubes.

**Patient concerns:**

A 7-year-old boy bedridden with hypoxic encephalopathy owing to drowning at the age of 1 year was admitted our hospital with urinary retention for 1 month. At the age of 4 years, a PEG-J tube was inserted. Concomitant with hyperaldosteronemia, an intestinal intussusception from the duodenum to the jejunum was observed via computed tomography (CT). The patient's condition worsened dramatically; gastrointestinal perforation was suspected, and laparotomy was performed.

**Diagnosis:**

Jejuno-jejunal intussusception.

**Interventions:**

Open surgery was performed to release the intussusception. By assessing the reduced intestinal tract, the intussusception starting from a 50 cm portion from the Treitz ligament had been extended to 100 cm from the Treitz ligament. The oral side jejunum was dilated. No evidence of intestinal perforation or strangulated ileus was observed, and the intussusception was manually remediable.

**Outcomes:**

Preoperative CT examination showed intussusception from the duodenum to the jejunum. Laparotomy showed intussusception on the anal side of the Treitz ligament. With regard to the CT findings associated with the progression of intussusception to the duodenal site, as a result of the telescope phenomenon extending to the duodenum due to the relaxation of the Treitz ligament through repeated intussusception, it was considered that CT examination revealed intussusception extending from the jejunum to the duodenum of oral side. After 3 postoperative weeks, the patient was finally able to return home.

**Lessons:**

If the ileus is observed during the insertion of a PEG-J, clinicians should consider the possibility of intussusception even in the duodenum.

## 1 Introduction

Percutaneous endoscopic gastrojejunostomy (PEG-J) tubes are used for enteral nutrition as percutaneous endoscopic gastrostomy (PEG).^[1]^ The tip of the tube, which is inserted by percutaneous gastrostomy, reaches the jejunum beyond the Treitz ligament. It is indicated for patients with gastrostomy, complications of gastroesophageal reflux disease and poor passage of nutrients from the gastric pylorus to the duodenum.^[2]^ Although PEG-J tubes are believed to reduce the side effect of aspiration, cautious catheter management is required.^[3]^

Intussusception owing to percutaneous endoscopic jejunostomy, PEG, or long ileus tubes has previously been reported in children and adults.^[4–6]^ Here, we discuss the pediatric case of jejuno-jejunal intussusception and telescope phenomenon to the inside of the Treitz ligament caused by a PEG-J tube.

## 2 Case presentation

A 7-year-old boy was admitted to our hospital with urinary retention of 1 month. He had experienced cardiopulmonary arrest owing to drowning at the age of 1 year and experienced hypoxic encephalopathy and was managed at home with a ventilator and feeding tube. PEG was performed at the age of 4 years, but the excretion from the stomach into the duodenum was exacerbated after PEG construction. Enteral nutrition was started using a PEG-J tube (MIC – TJ tube, 16 Fr, 45 cm; Halyard Healthcare, Inc. Yokohama, Japan). The tube was exchanged every 3 months at an outpatient clinic.

**Figure 1:**
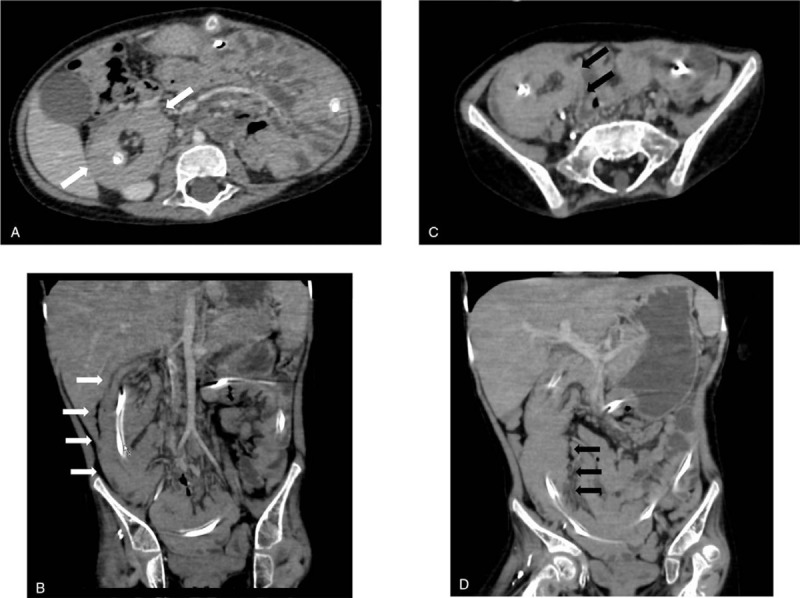
The contrast computed tomography scan showed wall thickening and intussusception of the duodenum (A, B; white arrows) and jejunum (C, D; black arrows).

**Figure 2:**
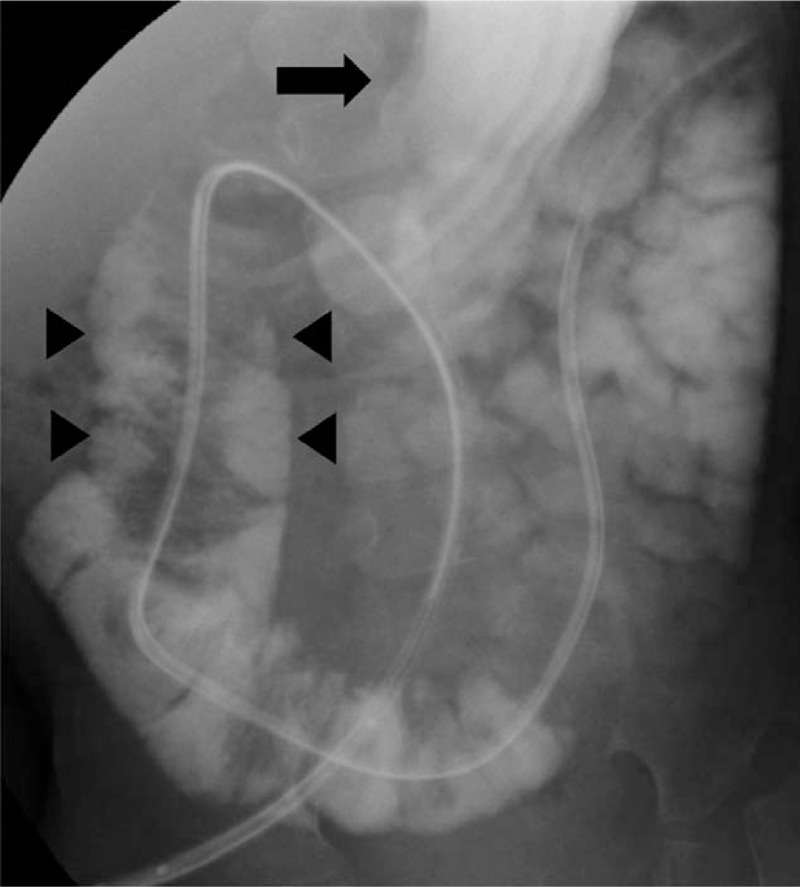
The upper gastrointestinal examination showed good passage from the stomach (arrow) to the duodenum (arrowhead).

**Figure 3:**
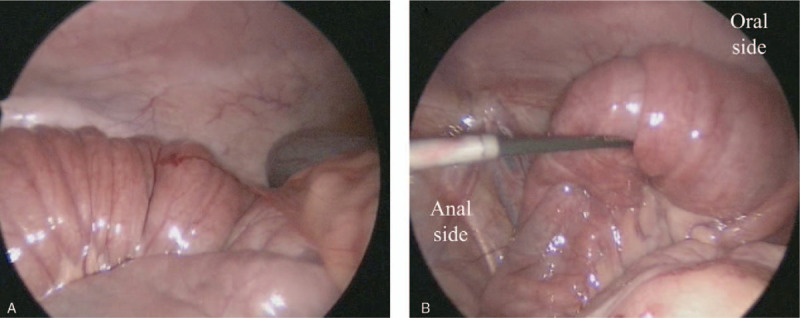
The small intestine at the site of intussusception presented a bellows structure and dilatation with a reddish and swollen appearance.

**Figure 4:**
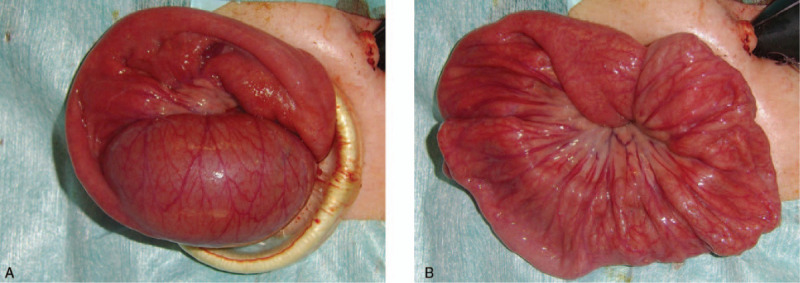
Operative findings of the small intestine. The intussusception starting from a 50 cm portion from the Treitz ligament had been extended to 100 cm from the Treitz ligament. The oral side jejunum was dilated (A). The intussusception was manually reduced by gentle proximal sequential and circumferential pressure (B). There was no evidence of intestinal necrosis or ischemia.

After admission, urinary retention was relieved by urinary catheterization; however, the patient's hyperreninemia, hyperaldosteronism, and hypokalemia (renin >20 ng/mL/h: normal range 0.2–2.3 ng/mL/h, aldosterone 1,110 pg/mL: normal range 29.9–158.8 pg/mL, potassium 3.0 mEq/L: normal range 3.6–4.9 mEq/L) continued. Contrast computed tomography (CT) scans obtained to identify the cause of hyperaldosteronism revealed wall thickening of the duodenum (Fig. 1A, B) and intussusception from the duodenum to the jejunum (Fig. 1A, B, C, D); however, upper gastrointestinal X-ray showed unobstructed passage from the stomach to the duodenum (Fig. 2). Two days later, convulsion and fever were observed; their cause could not be identified. Tazobactam/piperacillin and cefotaxime were administered, but almost no improvement in the patient's condition was observed. Black drainage fluid gradually appeared from the PEG-J tube. The patient's C-reactive protein (CRP) level increased to 25.3 mg/dL and he was transferred to the Department of Pediatric surgery at another hospital for emergency surgery due to suspected perforation. The diagnosis, based on the results of contrast CT, was strangulated ileus due to intussusception of the PEG-J tube, and an emergent operation was performed. Laparoscopic observation revealed a bellows-shaped intestinal tract with a reddish and swollen appearance from the bottom of the liver to the pelvis (Fig. 3A, B). Finally, open surgery was performed to release the intussusception and to observe the entire intestine. By assessing the reduced intestinal tract, the intussusception starting from a 50 cm portion from the Treitz ligament had been extended to 100 cm from the Treitz ligament. Intussusception findings was not clear from the Triz ligament up to 50 cm on the anal side. The oral side jejunum was dilated (Fig. 4A). No evidence of intestinal perforation or strangulated ileus was observed, and the intussusception condition was manually remediable (Fig. 4B). After surgery, the patient's CRP level declined gradually; hyperreninemia, hyperaldosteronism, and hypokalemia immediately improved. After 3 postoperative weeks, the patient was transferred to our hospital and was finally able to return home.

## 3 Discussion

This report presents a pediatric case of jejuno-jejunal intussusception and telescope phenomenon to the inside of the Treitz ligament caused by a PEG-J tube. There have been reports of cases in which a PEG tube caused intussusception at the duodenal site,^[7–10]^ but no reports of intussusception have been found to be caused by PEG-J tubes. In the present case, although preoperative CT showed intussusception from the duodenum to the jejunum, we initially thought that intussusception in the duodenum, which was fixed to the retroperitoneum, would not occur; thus, the diagnosis of intussusception with the PEG-J tube was delayed until the appearance of black drainage fluid from the PEG-J tube. Black drainage fluid was considered as gastrointestinal bleeding due to intestinal mucosal damage from intussusception. Preoperative CT examination showed intussusception from the duodenum to the jejunum. Laparotomy showed intussusception on the anal side of the Treitz ligament; this differed from preoperative CT findings. With regard to the CT findings associated with the progression of intussusception to the duodenal site, we considered that his main pathological condition was jejuno-jejunal intussusception, and the duodenal intussusception observed on CT imaging may have been a telescopic phenomenon.^[11]^ As a result of the telescope phenomenon extending to the duodenum due to the relaxation of the Treitz ligament through repeated intussusception, it was considered that CT examination revealed intussusception extending from the jejunum to the duodenum of oral side. The telescope phenomenon may not have been clearly observed during the operation. During the laparotomy, the telescope phenomenon may have been released.

After the intussusception was released by surgery, fever and CRP elevation quickly improved. It may have been a finding associated with disturbances in intestinal blood flow and infection. Seizures may have been triggered by stress associated with intussusception. As the cause of hyperreninemia, hyperaldosteronism and hypokalemia, pseudo-Bartter syndrome due to intravascular dehydration associated with intussusception was considered mostly. All abnormal findings before surgery improved quickly after reduction of intussusception.

It has been reported that the possibility of intestinal malrotation must be taken into consideration while managing children with intussusception.^[12–14]^ In our case, it seemed that the duodenum did not form a normal C-loop, and the position of the Treitz ligament was not normal on CT findings. Though we considered the presence of intestinal malrotation, CT showed no abnormalities in the small or large intestines. Brereton et al reported that intestinal malrotation has frequently occurred due to a lack of intestinal fixation, and it has been recommended to perform upper gastrointestinal imaging in all patients with intussusception.^[15]^ In our case, we suspected that the duodenum was ultimately dilated and extended due to repeated intussusception, not a complication of intestinal malrotation.

Intussusception by an ileus tube usually occurs 3 to 10 days on average after insertion^[16]^; intussusception more than 3 years after the insertion of a PEG-J tube is very rare. We cannot deny the possibility of repeated intussusception in the present case. Intussusception due to jejunal tube usage is said to be more common in cases involving the use of a pigtail tube, thicker tubes, or a long insertion length.^[17]^ The present patient had a body weight of 12 kg; thus, the PEG-J tube, which had a diameter of 16 Fr, was thick and may have triggered intussusception. PEG-J tubes may lead to complications such as intussusception, even if the duodenum is fixed to the retroperitoneum. In order to prevent intussusception caused by PEG-J tubes, it may be important to select a tube size that suits the physique, especially in children. During PEG-J tube placement, complications associated with tube should be considered for symptoms other than abdominal symptoms.

In conclusion, if the ileus is observed during the insertion of a tube such as a PEG, PEG-J, or ileus tube, clinicians should consider the possibility of intussusception, and even if a long time has passed since tube insertion. It is necessary to further investigate the epidemiology and cause of jejuno-jejunal intussusception and telescope phenomenon.

## Acknowledgments

The authors want to thank the patient and his family for their consent to write and publish this case report. The authors would like to thank Enago (www.enago.jp) for the English language review.

## Author contributions

**Conceptualization:** Toshihiko Kakiuchi.

**Data curation:** Toshihiko Kakiuchi, Aiko Nakayama, Junichi Nojiri, Takeshi Yamanouchi.

**Formal analysis:** Toshihiko Kakiuchi.

**Investigation:** Toshihiko Kakiuchi, Aiko Nakayama, Junichi Nojiri, Takeshi Yamanouchi, Muneaki Matsuo.

**Methodology:** Toshihiko Kakiuchi.

**Project administration:** Muneaki Matsuo.

**Supervision:** Junichi Nojiri, Takeshi Yamanouchi, Muneaki Matsuo.

**Validation:** Toshihiko Kakiuchi.

**Writing – original draft:** Toshihiko Kakiuchi, Aiko Nakayama.

**Writing – review & editing:** Muneaki Matsuo.

Toshihiko Kakiuchi orcid: 0000-0002-9995-5522.

## References

[1] El-Matary W Percutaneous endoscopic gastrojejunostomy tube feeding in children. Nutr Clin Pract 2011;26:78–83. 21266702 10.1177/0884533610392236

[2] Campwala I Perrone E Yanni G Complications of gastrojejunal feeding tubes in children. J Surg Res 2015;199:67–71. 26227672 10.1016/j.jss.2015.06.058

[3] Al-Zubeidi D Demir H Bishop WP Gastrojejunal feeding tube use by gastroenterologists in a pediatric academic center. J Pediatr Gastroenterol Nutr 2013;56:523–7. 23254445 10.1097/MPG.0b013e318282a8db

[4] Govednik C Cover J Regner JL Preventing retrograde jejunoduodenogastric intussusception as a complication of a long-term indwelling gastrostomy tube. Proc (Bayl Univ Med Cent) 2015;28:34–7. 25552793 10.1080/08998280.2015.11929179 PMC4264705

[5] Satoh T Sawada K Satoh M Small intestinal intussusceptions due to the placement of a percutaneous endoscopic jejunostomy tube. BMJ Case Rep 2011;2011: 10.1136/bcr.07.2010.3169 PMC3062045 22715249

[6] Ishii M Teramoto S Yakabe M Small intestinal intussusceptions caused by percutaneous endoscopic jejunostomy tube placement. J Am Geriatr Soc 2007;55:2093–4. 18081681 10.1111/j.1532-5415.2007.01439.x

[7] Ciaccia D Quigley RL Shami PJ A case of retrograde jejunoduodenal intussusception caused by a feeding gastrostomy tube. Nutr Clin Pract 1994;9:18–21. 8159136 10.1177/011542659400900118

[8] Alomari M Alomari A Hitawala A Anterograde gastroduodenal intussusception: a rare but lethal complication of percutaneous endoscopic gastrostomy tube placement. Cureus 2019;11:e4347 doi: 10.7759/cureus.4347. 31187012 10.7759/cureus.4347 PMC6541164

[9] Ibegbu E Relan M Vega KJ Retrograde jejunoduodenogastric intussusception due to a replacement percutaneous gastrostomy tube presenting as upper gastrointestinal bleeding. World J Gastroenterol 2007;13:5282–4. 17876902 10.3748/wjg.v13.i39.5282 PMC4171313

[10] Jamil Y Idris M Kashif N Jejunoduodenogastric intussusception secondary to percutaneous gastrostomy tube in an adult patient. Jpn J Radiol 2012;30:277–80. 22173561 10.1007/s11604-011-0036-5

[11] McGoon DC Intussusception: a hazard of intestinal intubation. Surgery 1956;40:515–9. 13360617

[12] Khan YA Yadav SK Elkholy A Waugh's syndrome: report of two children with intussusception. European J Pediatr Surg Rep 2017;5:e29–31. 10.1055/s-0037-1604264 PMC5533607 28761799

[13] Gil-Vargas M Sol-Melendez AK Miguel-Sardaneta ML Is intestinal malrotation the cause of intussusception? Waugh's syndrome, a case report. Cir Cir 2016;84:250–2. 26255768 10.1016/j.circir.2015.06.027

[14] Breckon VM Hadley GP Waugh's syndrome: a report of six patients. Pediatr Surg Int 2000;16:370–3. 10955564 10.1007/s003830000349

[15] Brereton RJ Taylor B Hall CM Intussusception and intestinal malrotation in infants: Waugh's syndrome. Br J Surg 1986;73:55–7. 3947878 10.1002/bjs.1800730123

[16] Okagawa Y Takada K Sakamoto H A case of intussusceptions at two parts of the ileum caused by an ileus tube. Nihon Shokakibyo Gakkai Zasshi 2017;114:1001–7. 28579583 10.11405/nisshoshi.114.1001

[17] Furuya Y Wakahara T Akimoto H A case of postoperative recurrent intussusception associated with indwelling bowel tube. World J Gastrointest Surg 2010;2:85–8. 21160855 10.4240/wjgs.v2.i3.85 PMC2999218

